# Alternative preparation of inclusion bodies excludes interfering non-protein contaminants and improves the yield of recombinant proinsulin

**DOI:** 10.1016/j.mex.2014.07.005

**Published:** 2014-08-12

**Authors:** Robert B. Mackin

**Affiliations:** Department of Biomedical Sciences, Creighton University School of Medicine, 2500 California Plaza, Omaha, NE 68178-0405, USA

**Keywords:** CNBr, cyanogen bromide, DTT, dithiothreitol, GSH/GSSG, reduced glutathione/oxidized glutathione, PS/DVB, polystyrene/divinylbenzene, RPC, reversed-phase chromatography, RP-HPLC, reversed-phase high-performance liquid chromatography, SDS-PAGE, sodium dodecyl sulfate-polyacrylamide gel electrophoresis, TCEP, tris(2-carboxyethyl)phosphine, TFA, trifluoroacetic acid, Improved purification of recombinant proinsulin, Proinsulin, Prohormone, Protein expression, Inclusion bodies, Protein purification, HPLC protein analysis

## Abstract

The goal of simple, high-yield expression and purification of recombinant human proinsulin has proven to be a considerable challenge. First, proinsulin forms inclusion bodies during bacterial expression. While this phenomenon can be exploited as a capture step, conventionally prepared inclusion bodies contain significant amounts of non-protein contaminants that interfere with subsequent chromatographic purification. Second, the proinsulin molecules within the inclusion bodies are incorrectly folded, and likely cross-linked to one another, making it difficult to quantify the amount of expressed proinsulin. Third, proinsulin is an intermediate between the initial product of ribosomal translation (preproinsulin) and the final product secreted by pancreatic beta cells (insulin). Therefore, to be efficiently produced in bacteria, it must be produced as an N-terminally extended fusion protein, which has to be converted to authentic proinsulin during the purification scheme. To address all three of these problems, while simultaneously streamlining the procedure and increasing the yield of recombinant proinsulin, we have made three substantive modifications to our previous method for producing proinsulin:.

• Conditions for the preparation of inclusion bodies have been altered so contaminants that interfere with semi-preparative reversed-phase chromatography are excluded while the proinsulin fusion protein is retained at high yield.
• Aliquots are taken following important steps in the procedure and the quantity of proinsulin-related polypeptide in the sample is compared to the amount present prior to that step.
• Final purification is performed using a silica-based reversed-phase matrix in place of a polystyrene-divinylbenzene-based matrix.

## Graphical abstract

**Figure:**
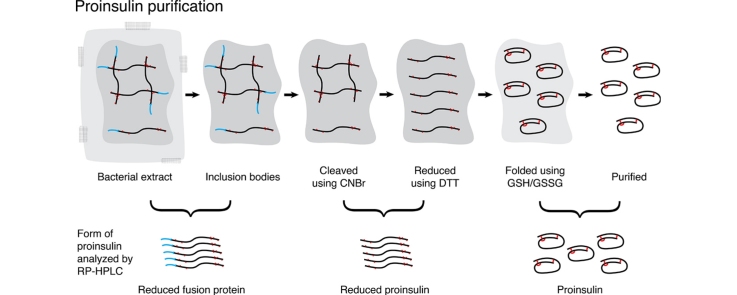

## Method details

### Step 1: proinsulin expression

The choice of using a prokaryotic versus a eukaryotic expression system for the production of a recombinant protein is frequently dependent on the target protein's folding requirements, the necessity of post-translational peptide bond cleavage and the presence or absence of other post-translational modifications (e.g. glycosylation, phosphorylation, sulfation, amidation, etc.). Production of proinsulin requires both protein folding with simultaneous formation of disulfide bonds (cysteine side chains and disulfide bonds are shown in red in the graphical abstract), and cleavage of a single peptide bond to remove an N-terminal extension peptide (the extension peptide is shown in cyan in the graphical abstract). However, proinsulin does not require any further post-translational processing and previous studies have demonstrated that proper folding, disulfide bond formation and cleavage of the N-terminal extension can be performed in vitro as part of the purification scheme [1]. Therefore, because of the time and cost savings provided by a prokaryotic expression system, *Escherichia coli* remains the organism of choice for heterologous expression of recombinant proinsulin.

#### Procedure

**Fig. 1:**
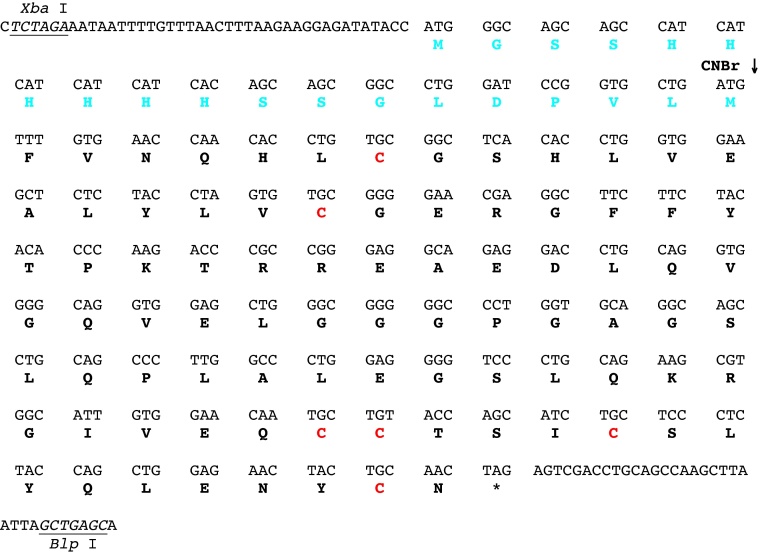
DNA and protein sequences of the fusion protein coding region. The restriction sites in pET-9b used for insertion of the indicated coding region are shown as underlined italic nucleotides, and the expressed fusion protein is indicated using one-letter amino acid abbreviations. The N-terminal extension peptide is shown in cyan and the cysteine residues involved in disulfide bonding are shown in red to match the color scheme used in the Graphical Abstract. The site where cyanogen bromide cleaves the fusion protein is indicated by CNBr ↓.

BL21(DE3) bacteria transformed with the plasmid hPI/pET-9b (both the insert cDNA and corresponding expressed protein sequences are shown in Fig. 1) were grown in LB-Miller medium and protein expression was induced by the addition of 0.4 mM isopropyl-β-d-1-thiogalactopyranoside once the bacteria reached an *A*_600_ of ∼0.8. The bacteria were cultured for an additional 2 h, collected by centrifugation (10,000 × *g* for 10 min at 4 °C), frozen on dry ice and stored at −80 °C. Volumes for all subsequent steps are given for the purification of proinsulin from 1 L of bacterial culture, which routinely generated an initial cell pellet of approximately 3.5 g (wet weight).

### Step 2: analytical RP-HPLC

**Fig. 2:**
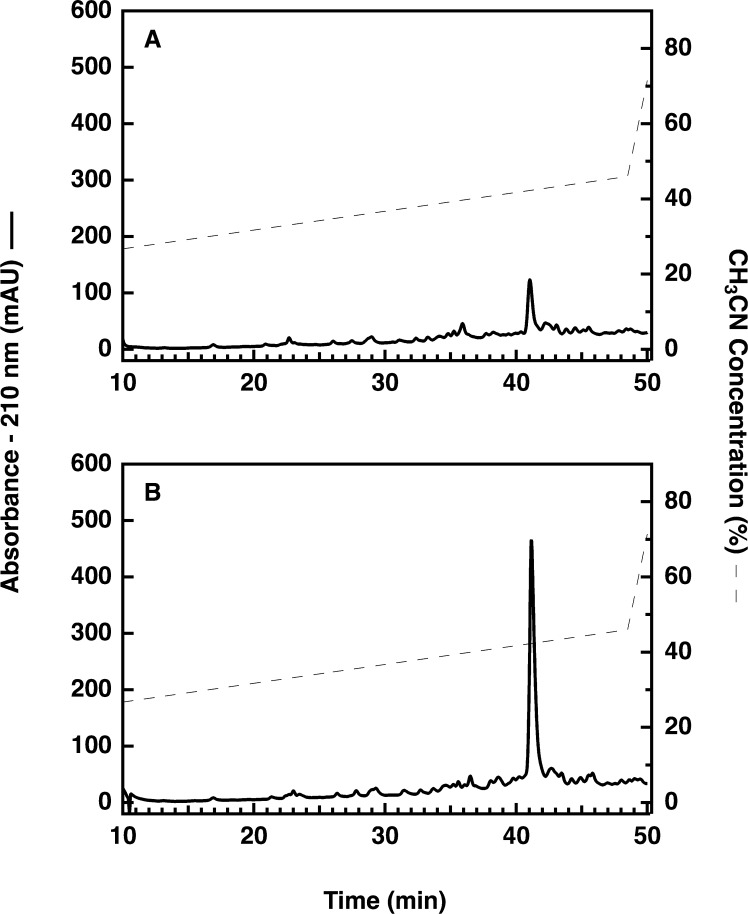
Effect of chemical reduction of samples prior to RP-HPLC analysis. The same size aliquot was analyzed in both panels. Panel A, the aliquot was analyzed without chemical modification. Panel B, the aliquot was reduced using TCEP prior to analysis. Detection of eluted proteins was performed at 210 nm and the peak at 41.2 min corresponds to reduced proinsulin fusion protein.

During experiments in which purification of proinsulin was initiated from 5 or 10 L of bacterial culture (using direct scaling from our previous 1 L procedure [1]), it was observed that the final yield of proinsulin did not increase in proportion to the starting culture volume. While investigating potential causes for this problem, we observed that at early stages of the purification procedure there was a significant difference in the integrated peak area for proinsulin-related peptides depending on whether or not the aliquots were chemically reduced prior to analysis by reversed-phase high-performance liquid chromatography (RP-HPLC). An example of these results is shown in Fig. 2 for aliquots from the initial bacterial extract.

During previous studies we had observed that the reduced and folded versions of both the fusion protein and proinsulin elute within the acetonitrile gradient used for the present analysis. This is of interest because the increase in size of the peak representing reduced fusion protein (41.2 min in Fig. 2A and B) is not matched by a decrease in size of another peak in this pair of chromatograms. The absence of a chromatographically identifiable, corresponding peak suggests that most of the fusion protein is present either as multiple incorrectly folded versions of the fusion protein, or as a disulfide-bonded oligomer containing multiple copies of fusion protein. A consequence of these observations is that it suggests that for over half of the purification steps (those preceding sample reduction with dithiothreitol) there are multiple forms of the target protein (reduced and incorrectly folded) in the sample (see graphical abstract).

As the target protein exists as two different polypeptides (fusion protein and proinsulin) and each polypeptide was anticipated to exist in two different chemical forms (reduced and folded) during the purification procedure, we were already facing the challenge of measuring the presence of four different forms of proinsulin-related polypeptide. It now appears that partially folded, misfolded or cross-linked versions of both forms of the polypeptide (fusion protein and proinsulin) could be present during proinsulin purification as well. If the non-chromatographically identifiable forms are referred to collectively as misfolded versions, the spectrum of potential forms of bacterially derived proinsulin-related polypeptide in a purification scheme increases to six: reduced, misfolded and folded versions of both the fusion protein and proinsulin. As we perform peptide bond cleavage and chemical reduction on the sample before attempting to re-fold proinsulin, one of these forms, folded fusion protein, is not expected to be present during the present purification procedure. So, in this specific purification scheme we would expect five different forms of the target protein.

To address the challenge of monitoring five different proinsulin-related polypeptides during the purification scheme, we attempted to simplify the complexity of the samples that would be analyzed. By performing a reduction procedure on samples that contain multiple chemical forms of the target polypeptide, we could reduce sample complexity down to three versions of the target protein: reduced proinsulin fusion protein, reduced proinsulin and folded proinsulin; with sample complexity at any individual step reduced to a single species of proinsulin-related polypeptide (see graphical abstract). This modification simplifies analysis and provides a means to monitor the recovery of proinsulin-related protein throughout the purification scheme.

#### Procedure

Aliquots of samples that needed to be chemically reduced prior to analysis (“bacterial extract-reduced” and “inclusion bodies-reduced” in Table 1) were brought to a volume of 120 μl with final concentrations of 2.5 M guanidine–HCl and 210 mM Tris/HCl at pH 8.7. The reducing agent Tris(2-carboxyethyl)phosphine (TCEP) was added to a final concentration of 7.5 mM and the sample was incubated for 30 min at room temperature. Iodoacetamide was then added to a final concentration of 25 mM and the sample was again incubated for 30 min at room temperature, but this time the incubation was performed in the dark. The reaction was stopped by adding 35 μl of glacial acetic acid to give a final sample volume of 180 μl.

Aliquots of samples that did not need to be chemically reduced prior to analysis (“bacterial extract”, “reduced proinsulin”, “folded proinsulin” and “purified proinsulin” in Table 1) were brought to a final volume of 180 μl using water.

For both the reduced and non-reduced samples, acetonitrile and TFA were added to give concentrations of 4% and 0.1% respectively, in a final volume of 200 μl. Aliquots (180 μl out of 200 μl) were then injected onto a 0.2 cm × 15 cm Jupiter C4 RP-HPLC column (5 μm particle size, 300 Å pore size, Phenomenex, Torrance, CA) equilibrated in 35% B (solvent *A* = 0.1% TFA in water; solvent *B* = 80% acetonitrile in 0.1% TFA in water). The column was run at 150 μl/min and, following a 5 min plateau at 35% B, peptides were eluted using a gradient from 35% to 55% B over the course of 30 min. Elution of polypeptides was monitored at 210 nm.

#### Quantification

Proinsulin concentrations were estimated using the integrated peak areas from analytical RP-HPLC. To do this, samples of previously purified proinsulin were weighed to one-tenth of a milligram, dissolved in 50 mM acetic acid and aliquots were analyzed using the above procedure. For our instrument (an HP 1090 liquid chromatograph with a diode-array detector) it was determined that chromatography of 1 μg of proinsulin resulted in an approximate integrated peak area of 3200 mAU s. Integrated peak areas (mAU s) from samples of unknown concentration were therefore divided by 3200 to calculate the number of micrograms of either reduced proinsulin fusion protein, reduced proinsulin or folded proinsulin in the sample.

While this calibration was only performed for the folded version of proinsulin, preliminary studies with the reduced version of proinsulin indicated that the instrument-specific extinction coefficient for this molecule is similar to the coefficient for the folded version. Determination of an extinction coefficient for the reduced version of the fusion protein was not possible as this protein was not available in purified form. It is expected that actual quantities of reduced fusion protein would be lower than determined using the extinction coefficient for folded proinsulin as the fusion protein contains 19 additional amino acids.

It should be noted that the integrated peak area method was not designed for absolute quantification of each potential form of proinsulin polypeptide. It was designed for use as a guide to the step-wise recovery of different forms of proinsulin polypeptide during the purification scheme. In this respect it has proven to be an extremely useful tool and has significantly aided our efforts to streamline the purification scheme and increase the overall yield of recombinant proinsulin.

### Step 3: preparation of inclusion bodies

**Fig. 3:**
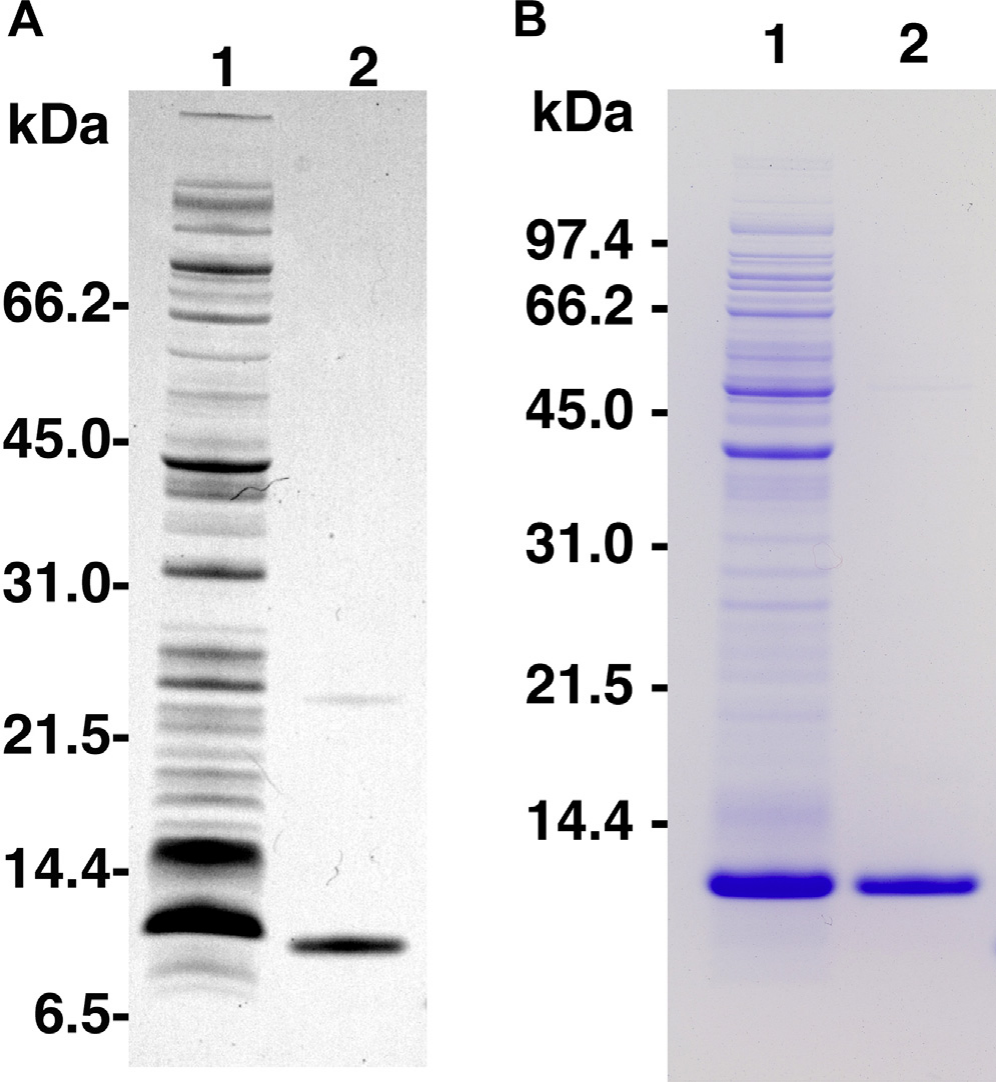
SDS-PAGE analysis of inclusion body preparation. Panel A shows the first two lanes of Fig. 3 from [1] (reproduced with permission), while panel B represents results from this study. In both panels, lane 1 is a sample of the bacterial extract while lane 2 is a sample of the prepared inclusion bodies. Proteins were separated on NuPAGE Bis–Tris polyacrylamide gels using MES running buffer. Panel A represents samples of DKP-hPI analyzed using a 4–12% gradient gel, while panel B represents samples of normal human proinsulin analyzed using a 10% gel.

During our previous study [1], we speculated that the contaminant responsible for the cloudy appearance of the sample prepared for reversed-phase chromatography (RPC) was one or more misfolded versions of proinsulin, and that their presence was an unavoidable consequence of the re-folding procedure. This hypothesis was consistent with the observation that there are very few contaminants in aliquots from early stages of the purification as judged by analytical RP-HPLC and sodium dodecyl sulfate-polyacrylamide gel electrophoresis (SDS-PAGE) (see Figs. 2 and 3, respectively, in [1]).

However, when we recently tested gel filtration as an alternative chromatographic procedure for purifying proinsulin, we observed additional UV peaks (280 nm) of magnitude similar to the peak representing proinsulin. We therefore began to suspect that non-protein contaminants in the inclusion body preparation (possibly elements of the cell wall remaining after incomplete digestion by lysozyme) might account for the cloudy contamination. We then reasoned that if the contaminants were not proteins they should not be integral components of the purified inclusion bodies and that it might be possible to remove them using an improved method for inclusion body preparation.

While minor modifications have been adopted to facilitate the purification of specific target proteins, the standard approach for the preparation of inclusion bodies has remained the same for almost twenty years [2,3]. The standard method involves the lysis of bacteria cells using any of a variety of methods, followed by multiple rounds (usually 4 or 5) of high speed centrifugation (20,000 × *g* for 20 min at 4 °C) combined with re-suspension and washing of each pellet using mild detergent- and/or chaotropic agent-containing solutions. We reasoned that as much of the proinsulin fusion protein is likely to be cross-linked in a disulfide-bonded matrix (see Step 2 for discussion), and the non-protein contaminants would likely be less dense than the protein contents, it might be possible to use fewer centrifugation steps at lower speed and higher temperature to separate the proinsulin in the pellet from the non-protein contaminants in the supernatant. Results shown in Fig. 3 suggest that these hypotheses are correct.

#### Procedure

The frozen *E. coli* cells were thawed, re-suspended in 18 ml of BugBuster, 18 μl (450 units) of Benzonase and 0.6 μl (18,000 units) of Lyse-Aid rLysozyme. (BugBuster and Benzonase were from EMD Millipore, Billerica, MA and Lyse-Aid rLysozyme was from Semba Biosciences, Madison, WI). The suspension was gently shaken at room temperature for 20 min, subjected to approximately 15 s of homogenization using a Tissue Tearor homogenizer set at full speed (BioSpec Products Inc., Bartlesville, OK, setting “35”) and aliquots were taken for analysis. The sample was centrifuged at 8000 × *g* for 15 min at 20 °C and the supernatant was removed and discarded. The pellet was re-suspended in 18 ml of 50 mM Tris/HCl, pH 8.0, 50 mM NaCl, 0.5 mM EDTA, 5% glycerol and homogenized using the same conditions as above. The sample was again centrifuged at 8000 × *g* for 15 min at 20 °C and this supernatant was also removed and discarded, leaving behind the inclusion body pellet.

### Step 4: cleavage of the fusion protein, reduction and refolding of proinsulin

Because proinsulin is a biosynthetic intermediate and does not possess the N-terminal methionine residue necessary to initiate translation, when expressed in bacteria the recombinant form of this protein must be translated as a fusion protein whose N-terminal extension provides the initiator methionine. In the present purification scheme a poly-histidine affinity tag is present at the N-terminus. In spite of not being used for affinity purification, this tag is advantageous because, at 19 amino acids in length, it provides us with an easily observed indicator, using either RP-HPLC or SDS-PAGE, that the N-terminal methionine (along with the rest of the poly-His affinity tag) has been removed from the fusion protein.

(*Note*: while a previous study indicated that proinsulin is subject to non-specific N-terminal proteolytic degradation in some bacterial expression systems and that some N-terminal extensions decrease the rate of degradation [4] (another reason we chose the rather lengthy poly-His affinity tag as the fusion partner), we have not observed proteolytic degradation with either affinity-tagged N-terminal fusion proteins or single methionine residue-extended forms of proinsulin.)

The combined consequence of the expression of an N-terminally-extended fusion protein with a target protein containing six cysteine residues is that in a prokaryotic expression system the purification scheme is complicated by the potential presence of six different forms of the target protein (see discussion in Step 2). While we have narrowed the number of potential forms in the current purification scheme to five, the following multi-step procedure is important because it efficiently converts the expressed proinsulin fusion protein (which exists in two chemical forms) into the single desired product, folded proinsulin.

#### Procedure

The inclusion body pellet (∼0.35 g) was suspended in 2 ml of water, resulting in an opaque, muddy brown solution. To this, 8 ml of 88% formic acid was added, resulting in a 10 ml sample containing 70% formic acid that is completely transparent. Aliquots were taken for analysis and then approximately 0.42 g of cyanogen bromide (CNBr, final concentration = 400 mM) was added to the sample. The sample was placed in the dark in a chemical fume hood, and the reaction was allowed to proceed overnight at room temperature. The next morning, both the CNBr and formic acid were removed by rotary evaporation, leaving a white residue in the flask.

The residue was solubilized in 10 ml of 6 M guanidine/100 mM HCl, and then Tris was added to a final concentration of 500 mM, resulting in a solution of pH 8.1. Dithiothreitol was added to a final concentration of 50 mM, the sample was allowed to sit at room temperature for 60 min and aliquots were taken for analysis.

The reduced sample was subjected to buffer exchange using a 2.5 cm × 26 cm column of Superdex G-25 Superfine (GE Life Sciences, Piscataway, NJ) equilibrated in 50 mM glycine/NaOH, pH 10.5, 1 mM EDTA. The buffer exchange was performed at 2.0 ml/min and the protein peak was collected based on UV absorbance at 280 nm. The recovered, reduced proinsulin was diluted to 125 ml (approximate final concentration of 100 μg/ml) using fresh 50 mM glycine/NaOH, pH 10.5, 1 mM EDTA. Reduced and oxidized glutathione (Sigma-Aldrich, St. Louis, MO) were added to a final concentration of 1 mM each, the sample was briefly bubbled with argon, the reaction container was sealed and the sample was incubated overnight at 4 °C.

### Step 5: semi-preparative reversed-phase chromatography

**Fig. 4:**
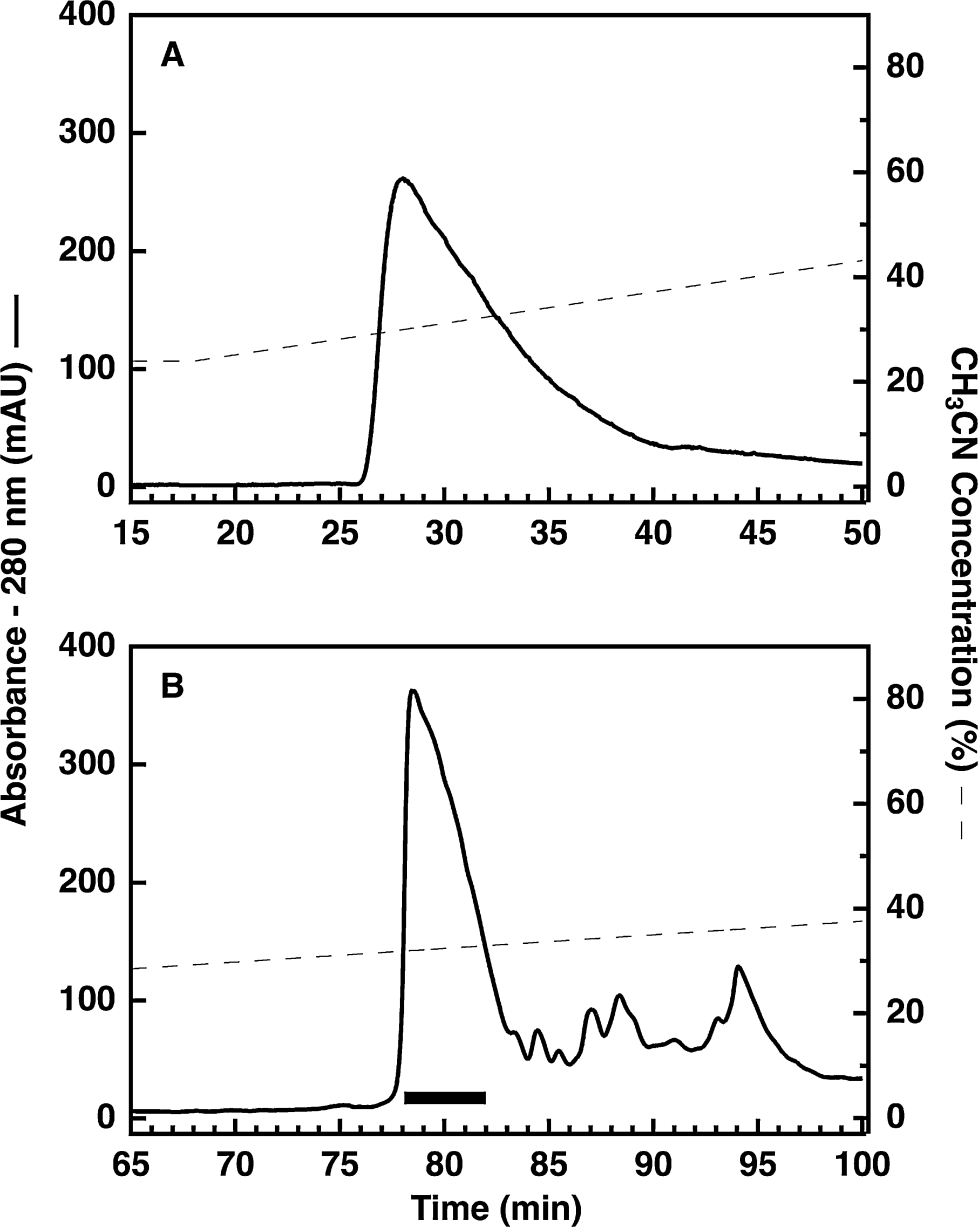
Comparison of different reversed-phase matrices for reversed-phase chromatography. Panel A, semi-preparative RPC purification was performed using a PS/DVB-based SOURCE 15RPC column (see [1] for details). Panel B, semi-preparative RPC purification was performed using a silica-based Jupiter C4 column. Detection of eluted proteins was performed at 280 nm and the fractions combined for further analysis, and future experiments, are indicated by the pooling bar in panel B.

Having previously developed a chromatographic method for the purification of proinsulin using a polystyrene-divinylbenzene (PS-DVB) matrix for semi-preparative reversed-phase chromatography, we hoped to migrate our method to a silica-based matrix following the adoption of our improved method for the preparation of inclusion bodies. While not a barrier to successful purification, use of the PS-DVB media resulted in significant peak tailing that required us to screen 14–16 fractions, to identify the 5 or 6 fractions that contained proinsulin of acceptable purity (for example see Fig. 4A).

**Fig. 5:**
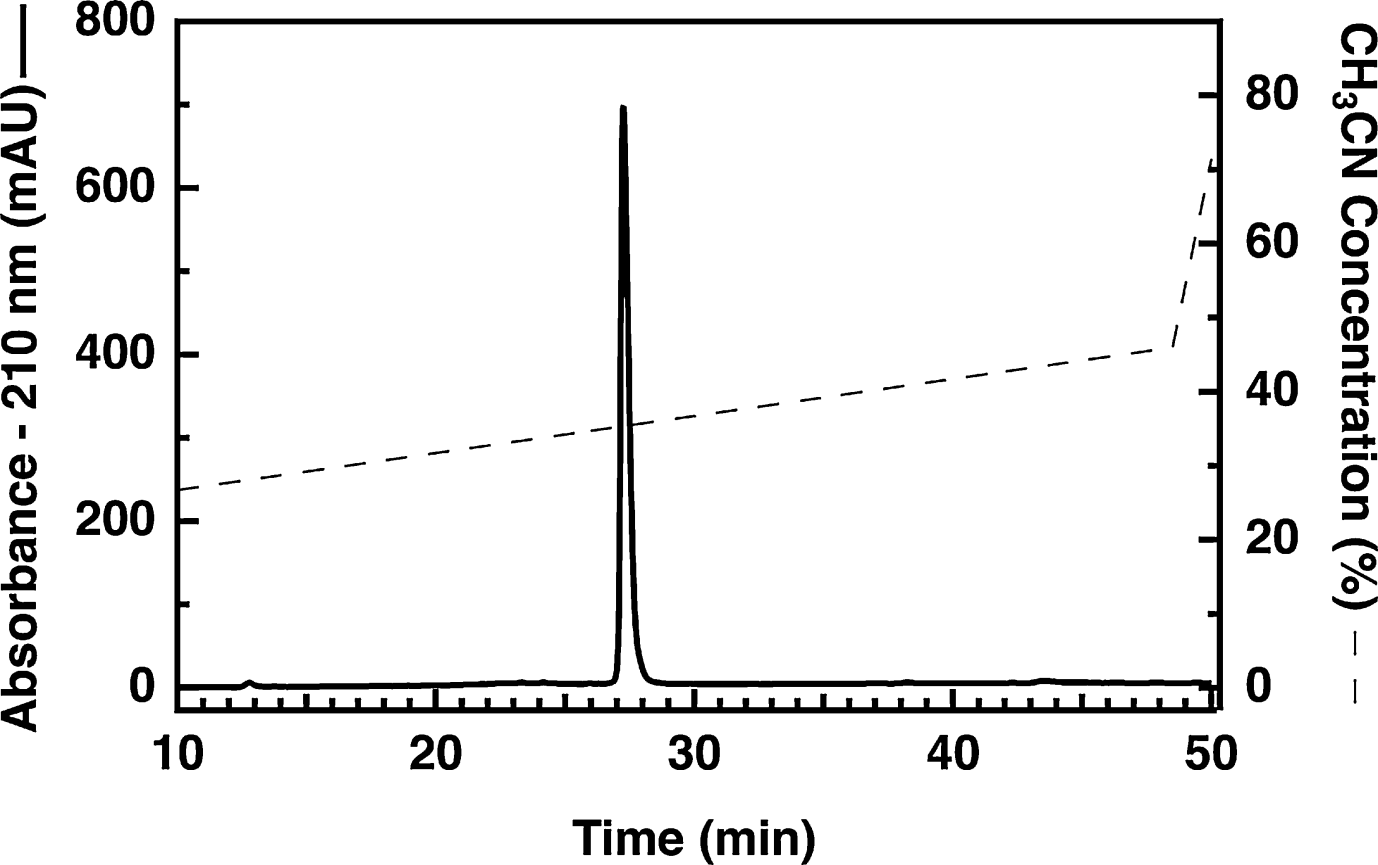
Analysis of purified proinsulin following reversed-phase chromatography. Approximately 5 μg of PI was loaded onto a Jupiter C4 RP-HPLC column. Detection of eluted proteins was performed at 210 nm and the peak at 27.2 min corresponds to folded proinsulin.

Previously, the use of silica-based particles was not possible because samples of folded proinsulin originating from our old inclusion body preparation became cloudy when we acidified them to pH 2.0 for RPC. Fortunately, when samples generated using our new method for inclusion body preparation were acidified the samples remained clear, and when these samples were filtered the proinsulin passed through the filter membrane. We therefore examined the use of a conventional, silica-based chromatography column for semi-preparative RPC. Compared to the PS/DVB-based RPC column we had been using, we observed both a sharper peak shape and the separation of contaminants away from proinsulin (see Fig. 4B). In addition, there was no increase in column back-pressure during sample loading, as we had observed in our previous study [1]. Finally, we are now able to screen 6 or 8 fractions to identify the 3 or 4 fractions that contain purified proinsulin. As shown in Fig. 5, the purity of proinsulin prepared starting with the modified inclusion body technique is excellent (∼99% by UV absorbance at 210 nm) and the overall yield (routinely greater than 15 mg per L of bacteria) is approximately three times higher than obtained using our previous method [1].

#### Procedure

Following the refolding procedure, aliquots (25 μl) were taken for analysis to ensure that efficient folding had taken place. The rest of the sample was then prepared for semi-preparative RPC by bringing the solution to final concentrations of 0.1% trifluoroacetic acid (TFA), 4% acetonitrile, and 55 mM HCl in a final volume of 166 ml. The sample, now at pH 2.0, was filtered through a 0.45 μm pore size polypropylene depth filter (Whatman Puradisc 25 PP, GE Life Sciences) and divided into two 83 ml aliquots. The first 83 ml aliquot was injected onto a 1.0 cm × 25 cm Jupiter C4 column (15 μm particle size, 300 Å pore size, Phenomenex) equilibrated in 35% B at a flow rate of 2.0 ml/min (solvent *A* = 0.1% TFA in water; solvent *B* = 80% acetonitrile in 0.1% TFA in water). Proinsulin was eluted using a gradient of 35–48% B over the course of 40 min. Elution was monitored by UV absorbance at 280 nm and 1.0 min fractions were collected. The second 83 ml aliquot was then purified using the same procedure. Fractions from each chromatography run were evaluated using analytical RP-HPLC and the appropriate fractions were pooled together (Fig. 4B). Aliquots were taken for analysis and the rest of the sample was then lyophilized.

#### Quantification

Our success at developing a method for proinsulin quantification and purification is summarized in Table 1, where the amounts of both proinsulin and total protein, along with sample volumes and aliquot sizes taken for RP-HPLC analysis, are shown for multiple steps in the purification scheme. (total protein concentration was determined using the Pierce BCA – Reducing Agent Compatible protein assay kit with bovine serum albumin as standard (Thermo Fisher Scientific, Inc., Waltham, MA)). Interestingly, the amounts of proinsulin (17.8 mg) and total protein (13.1 mg) in the “Purified proinsulin” sample are more different than we would expect for a protein with an estimated purity of 99% (Fig. 5). We hypothesize that either the BCA total protein assay underestimates the amount of proinsulin (represented as total protein in the “purified proinsulin” sample), or that the lyophilized proinsulin weighed out and used as a standard when determining the HPLC extinction coefficient contained non-proinsulin material (likely salts) that affected the calibration.

Fortunately, this discrepancy does not adversely affect the utility of either our analytical procedure or the purification scheme. As mentioned in step 2, the analytical procedure was designed as a convenient method for monitoring the amount of proinsulin-related polypeptide recovered following individual steps of the purification procedure. In this respect it was successful because it revealed that our previous procedure [1] resulted in low recovery of folded proinsulin relative to the amount of combined misfolded and reduced fusion protein present in the bacterial extract. Previously, based on the amount of just the reduced fusion protein in the bacterial extract, we were misled into thinking that 5 mg of proinsulin represented high recovery of this particular target protein. Once poor recovery early in the purification scheme was identified as a problem, the assay allowed us to improve both the inclusion body preparation procedure and the RPC step. The net result of these steps was the development of an improved procedure that significantly increases the overall yield, and purity, of recombinant proinsulin.

## Figures and Tables

**Table 1:** Purification yield.

Aliquot	Proinsulin^a^ (mg)	Total protein^b^ (mg)	Sample volume (ml)	Aliquot volume (μl)
Bacterial extract	7.6	–	18	3
Bacterial extract-reduced	38.1	205.0	18	3
Inclusion bodies-reduced	32.2	62.9	10	2
Reduced proinsulin	29.9	47.8	10	2
Folded proinsulin	21.5	40.0	125	25
Purified proinsulin	17.8	13.1	16	5

a Calculated from the integrated peak area at 210 nm during analytical RP-HPLC.

b Measured using the BCA-Reducing Agent Compatible protein assay kit with bovine serum albumin as the standard protein.

## References

[1] Mackin R.B., Choquette M.H. (2003). Expression, purification, and PC1-mediated processing of (H10D, P28K, and K29P)-human proinsulin. Protein Expr. Purif..

[2] Nguyen L.H., Jensen D.B., Burgess R.R. (1993). Overproduction and purification of sigma 32, the *Escherichia coli* heat shock transcription factor. Protein Expr. Purif..

[3] Palmer I., Wingfield P.T. (2012). Preparation and extraction of insoluble (inclusion-body) proteins from *Escherichia coli*. Curr. Protoc. Protein Sci..

[4] Talmadge K., Gilbert W. (1982). Cellular location affects protein stability in *Escherichia coli*. Proc. Natl. Acad. Sci. U. S. A..

